# Mental Health Literacy and School-Student-Family Alliance: A Brief Narrative Review of Canada’s Individualized Education Plan

**DOI:** 10.7759/cureus.41894

**Published:** 2023-07-14

**Authors:** Xuechen Yuan

**Affiliations:** 1 Department of Education, Lakehead University, Thunder Bay, CAN

**Keywords:** special education, person-centred care, exceptional students, strength-based, mental health literacy, individual education plan

## Abstract

This review article addresses the historical context of power dynamics in individualized education planning processes for exceptional students within Canada's K-12 (i.e., from kindergarten to grade 12) education system. It highlights how such dynamics have created conditions for educators' internalized bias toward students with special needs. This article presents empirical evidence supporting the effectiveness of the strengths-based approach in individualized education planning and documentation to reshape the school-educator-student alliance and accomplish special education goals, emphasizing the importance of incorporating input from exceptional students and their families, advocating for students' self-determination, and shifting away from the traditional pathological approach. This article calls for future research on strengths-based approaches, mental health literacy, and post-school transitions while addressing multiscalar barriers using an intersectional lens.

## Introduction and background

In Canada, research involving special education of exceptional students in the K-12 (i.e., from kindergarten to grade 12) curriculum has extensively focused on developing and enacting procedures for student accommodation plans. However, the current understanding of special education remains incomplete because only a few previous works have examined the implementation of accommodation plans and their impact on students’ subsequent academic progress [1,2]. One of the legal documents in Canada named the individualized education plan (IEP) demonstrates a set of schools’ standards to assist exceptional students’ needs in working toward meaningful academic and lifestyle transition and personalized goals [2]. However, beyond the document’s capacity is the issue of power dynamics produced in its implementation in schools; as Sensoy and DiAngelo [3] mention that “even people who support mixed classes often do so to ‘help’ children with disabilities, assuming that the flow of knowledge and benefit is always from the able-bodied to the disabled” (p. 108). As educators may not be aware of their unearned privileges, recent studies have shown that IEP processes struggle to realize that students and their parents would have equitable access to the decision-making process compared to education experts [1,2,4]. Geltner and Leibforth [5] suggest that the language (jargon) and its underlying connotation (neutral) in IEPs for conceptualizing exceptional students’ educational challenges are too often deficit-oriented. This unequal distribution of power ultimately leads to a disconnection between the initial purpose of the IEP and its benefits on students’ academic achievement [6,7].

This review article aims to reevaluate the IEP content development and implementation in Canada's K-12 curricula while introducing a strengths-based framework that enhances exceptional students’ representation and successive participation in IEP processes [8]. The review focus will be addressing the specific question: How might strengths-based IEPs transcend barriers to relationship development between exceptional students and educators in accomplishing special education goals in Canada's K-12 educational system?

By shedding insight into power dynamics in educational institutions, this article may set the basis for school staff to look into their unconscious biases and transform IEPs as a legal requirement into a more genuine development process led by exceptional students and their families [3].

## Review

Methodology

This study is a brief narrative review that focuses on selecting articles directly addressing the topic of deficit-oriented and strengths-based approaches in IEPs. The databases used for the article scan are Google Scholar, PubMed, and PsycINFO. The search criteria used in this review identified a preliminary count of 30 articles by using terms like "individualized education plan," "individual education plan," "mental health literacy," "strengths-based IEP," and "IEP Canada," which include policy briefs and ministry webpages. All articles were written in English in the context of European and North American contexts. Most articles selected were conducted within the last 15 years, including case studies, qualitative interview studies, policy recommendations, and review articles. Three articles that predominantly focused on legal frameworks were excluded as they were out of this study's scope. Articles written in the global context aside from Canada were also reviewed due to a shortage of articles in the Canadian context. Scholarly works including books (*N* = 4) indirectly related to the topic were reviewed for providing critical insights into the social construction of disability and wellness. The final count of the literature reviewed was 31.

Background of IEP in Ontario, Canada

IEP is a contextualized document adopted by Canada and the US as the standard protocol of special education [7,8]. The development of similar legal documents supporting the needs of special education also emerges in many other countries' educational legislative frameworks [9,10]. Within the Canadian context, IEP implementation is contingent on the legal situations of provincial governments instead of the federal government, as section 93 of the Constitution Act, of 1867 states that the “federal government of Canada detains no constitutional authority” [11]. In 2000, the Ontario Ministry of Education outlined an IEP template that describes mandatory requirements of creating personalized programs for each student who is identified as exceptional by the Identification, Placement, and Review Committee, in compliance with Regulation 181/98 [12,13]. The committee usually comprises at least three members: school board members, teachers, and other special education experts [14]. They work closely with parents and the student (if available) to assess the student’s needs during IEP meetings before determining their exceptionality and appropriate level of support [14,15]. Upon agreement on the final decision between parents and the committee, the school will develop a written IEP based on observations and evaluations made by the committee. The document must then be presented to the student, their parents, and IEP teams [16].

The standard way for creating IEPs consists of a set of guidelines for describing the current level of student achievement, "annual program goals, learning expectations, teaching strategies, and assessment methods” [13]. Students’ progress in IEPs will be continuously monitored, reviewed through annual Identification, Placement, and Review Committee meetings, and then be reported to parents, allowing school teams to make further adjustments to the plan and ensure the appropriate compliance with learning outcomes in IEPs. Furthermore, under Ontario Regulation 181/98, transition planning is mandatory for each student over 14 years old [12]. When an exceptional student approaches the transition to future work and education, the school principal plays a primary role in referring to community service partners and consulting with post-secondary educational institutions, as well as identifying attainable goals to ensure affluent transitions [8,12].

Current problems with IEP

The understanding of people with special needs is rooted in the social construction of normality and abnormality [17], through which clinical practices, education, and social services sustain stigmatized and tabooed perceptions of mental illness [18]. People deemed exceptional nowadays would have been stigmatized as “feeble-minded” in Canada around the early 1900s [3]. Those classifications were predominantly supported by eugenic policies, involving coercive sterilization and segregation of "othered" individuals from social institutions to rationalize the systemic privilege of dominant groups [3]. The emergence of neoliberalism that has shaped Canada's education reform over the past three decades leads to the commodification of social welfare and public health, which enables dominant groups to continue internalizing their preconceived power by ascribing their superiority to individual merits such as personal liberation and able-bodiedness while denying their unearned structural privileges [19]. While they rarely admit as much, dominant groups often take for granted that marginalized individuals must work harder and receive assistance from adept people to overcome their barriers.

In special education contexts, the rationalization of being able-bodied often reflects teachers’ subjective judgments toward exceptional students. Despite their efforts to overcome cognitive bias when evaluating students’ capacity, teachers inevitably hold specific expectations for students based on socially desirable characteristics [3,17]. Multiple articles have highlighted the concerns for deficit-oriented connotations in current IEPs and educational assessments, often created by education experts to undermine exceptional students' individual uniqueness, structural challenges, and chances for growth [7,18,20]. This has been shown to reduce the potential for partnership between educator-student-family in determining and following up with goals outlined in IEPs [21,22].

Concerning language construction in developing IEP documents, it is not uncommon for the Identification, Placement, and Review Committee to depict students’ special education status based on a traditional pathological focus [8]. Such representation usually involves a neutral connotation and a passive voice that alludes to exceptional students as social deviants from school standards and passive special education recipients [7]. For example, Elder et al. [20] demonstrated a vignette of what a deficit-oriented proposal might entail:

Franklin is a second-grade student who has labels of attention deficit/hyperactivity disorder (ADHD) and an intellectual disability. Franklin often has difficulty staying on task and focused. He also has a hard time comprehending and recalling material. Currently, Franklin is on grade level for math, but is well below grade level in reading. Specifically, Franklin has a hard time comprehending and recalling information read from a text. Because of these difficulties, Franklin is often unable to recall information from both independent reading books and books read aloud. [20]

Problematic words, such as hyperactivity, disability, hard time, well-below, difficulty, and unable, are substantial evidence that illustrates how judgmental characteristics in neutral IEP discourses inform one's internalized privileges. Furthermore, using passive voices could further establish dominance over students, as illustrated in statements extended by "it was suggested," '"o be modified," and "was recommended." Not only does such language construction absolve educators from their responsibilities, but it also enables them to be portrayed as actively controlling students' educational futures [7]. Meanwhile, such a unilateral relationship may actually increase the burden on educators in IEP creation and implementation as they may feel like "doing everything for them."

Simultaneously, while it is true that students’ parents may agree with what is proposed in IEP documents, their contributions to the decision-making process of children’s IEPs remain minimal [1]. Geltner and Leibforth [5] argued that parents often report feelings of alienation due to the jargon used in the process and school staff showing insufficient respect regarding parental contributions. Underlying reasons point toward school staff’s authoritative power in reinforcing the conception that individuals at the lower end of the relational power continuum will contribute less or no valuable insight into conventional meetings.

Since the relationship development in IEPs continues to be unidirectional, many critical aspects in special education, most importantly, the requirement of designing students’ long-term transition goals, are commonly disintegrated from IEP procedures [21]. It is also evident that IEPs for students with more emotional, learning, and behavioral challenges tend to lack sufficient transition support owing to biases and negative assumptions toward students with specific cultural phenotypes and exceptions [23]. IEP is also commonly recognized by research as a weak part of special education initiatives, given that the IEP documents are not really individualized with illy defined plans and assessments of learning, this has led to educators being skeptical of the real benefits of IEP implementations [2].

Recommendations for strengths-based approaches

Counseling fields were dominated by a pathological focus on individual needs, up until the mid-1990s, when strengths-based practices emerged. Strengths-based approaches concentrate on individual factors (e.g. skills, perspectives, and talents) that might act as a major incentive for self-empowerment [8]. This approach mitigates the lack of attention on identifying students’ skills in IEP processes and makes strategies that advocate for positive reinforcement instead of problem prevention [5].

Educators play a substantial role in daily interaction with young people who might require mental health support. However, many educators have not obtained professional training in mental health instruction for acknowledging various students’ needs and needs for trauma-informed approaches. Gregory [24] found that pre-service educators perceived their training in teacher education programs as insufficient for them to understand special needs holistically and partake in inclusive education. Frequently, insufficient preparation for mental health literacy might further augment students’ stigma and reduce coping skills for emotional disturbance [25]. In response to this dilemma, the University of British Columbia in Canada developed a mandatory curriculum, “Teach Mental Health,” to prepare all pre-service teachers with sufficient mental health knowledge for classroom integration and fulfill university graduation requirements [25]. There have been other existing nationwide anti-mental-stigma initiatives developed in the context of Canada including HeadStrong and Mental Health First Aid developed by the Mental Health Commission of Canada and the Talking about Mental Illness program which involves education, outreach, and advocacy pieces to inspire diverse student voices of mental health issues, address strategies for mental well-being, and enhance mental health knowledge of all Canadians [26].

Additionally, Carr and Frank [25] emphasized the importance for educators of using mental health terminology accurately and appropriately in everyday interactions to not exaggerate thoughts associated with mental illness. Individuals must also critically evaluate the results of medical studies and the jargon used to distract authentic opinions. Improving mental health literacy to leverage students’ self-efficacy in their health outcomes could reflect positive attributes used in strengths-based IEP processes [8]. Strengths-based practices offer a paradigm shift in our perception of thinking about negative behaviors.

Weishaar [8] demonstrated that it is imperative to develop critical thinking literacy in strengths-based IEP processes, in which students’ negative behaviors should be conceptualized as behavioral needs rather than deficits in their personalities. For example, when students display cognitive difficulties, rather than saying that “they are unable to solve the task," an indication of intellectual deficits, rephrasing it with “they require more scaffolding to solve the task” provides a more realistic assessment of the student’s current progress. This also motivates both students and educators to align with working on actionable educational planning as an indication of students’ present capacities. A strengths-based educational assessment would look like this, according to Elder et al. [20]:

Franklin is a friendly young boy who enjoys trains and tall buildings. Franklin is timid in new situations but warms up to people quickly. He is a hard-working second grade student who enjoys attending school, working with his teachers, and developing relationships with peers. He enjoys and excels in math. During math instruction, he likes to use manipulatives when working to solve a given problem. Currently, Franklin is working at mastering double-digit addition problems. When given 10 double-digit addition problems, Franklin gets an average of six correct. However, when given assistance, such as the teacher drawing a line between the two-digit number, Franklin is able to solve them correctly most of the time, as long as there are no carryovers. Franklin has labels of intellectual disability and ADHD that affect him academically because it is more difficult for him to comprehend and remember material, and his label of ADHD makes it harder for him to stay on task and focus for the duration of a lesson. These challenges are often evident during reading instruction. Specifically, he often has difficulty recalling information from both independent reading books at the first-grade level. For example, when prompted by saying, “Did _________ happen in the story?” Franklin often guesses, it is unclear if he comprehended what occurred in the story. However, when Franklin is given a graphic organizer to write down key aspects of the story and a peer buddy, he is able to more easily recall and pull out relevant textual information. Further, when these supports are paired with a text of his choice, he is able to pull out key events and details of the text with little adult assistance and he is able to recall information from the text more consistently. [20]

Blackwell and Rossetti [1] argued that students’ participation in IEP meetings and implementation is often a passive process, even though the importance of self-expression and goal determination in learning and transition has been widely acknowledged. They also find that instructing students to create their own IEPs would lead to better academic achievement than preparing it for them. Obtaining this occurs by constructing self-awareness and a sense of entitlement when students draw inferences about their characteristics, preferences for instructions, and attainable goals [27]. Furthermore, student-led IEPs yield more opportunities for educators to provide scaffolding based on students’ present progress for more accurate instructional and transition planning. For example, a student who wishes to improve counting skills from 1 to 99 would actively involve themselves in developing smart goals by leveraging their strengths, be it pattern recognition skills, musical and rhythmic skills, or spatial reasoning ability. Then, teachers could create preferable methods based on these self-identified assets. Student-led IEPs may also potentially alleviate the workload of educators by distributing the responsibility of creating and implementing legal documentation which would otherwise fall solely on educators as the sole authority [28].

Furthermore, understanding exceptional students’ educational needs will remain incomplete if IEP processes do not gauge families’ perspectives, concerns, and expectations in creating legal documents for school professionals. Therefore, a change in the atmosphere of IEP meetings and subsequent family support demands a critical shift in parents’ role from merely consent providers to agents of self-determination [29]. Encouraging school-family alliances entails transcending barriers of communication, convenience, and internalized inferiority so that parents will have greater access to expert knowledge and subsequent decision-making [3]. Thus, the first recommendation for the educator-family alliance is for school administrators to initiate personal connections with students’ families before the planning meeting. Parents require a preview of procedures and discussions that will take place during meetings, and additional guides on how IEP components are determined. In making this suggestion, it would be imperative to have a designated IEP member for administering this contact.

The next recommendation focuses on family involvement in developing collaborative IEPs to gather students’ histories, strengths, instructional goals, and future transitions during initial and follow-up meetings. Keyes and Owens-Johnson [21] found a person-centered approach highly useful when used in conjunction with satisfying IEP procedural requirements. For example, a person-centered approach helps understand educational expectations and lifestyle choices through inquiry and self-advocacy of students’ many talents, deep-seated desires, and unique life experiences. Consequently, these valuable observations would aid IEP team members to construct strengths-based plans for personalized learning objectives [21]. Person-centered planning meetings encourage students and their parents to disclose their needs, goals, dreams, and nightmares through information-gathering processes while still complying with IEP procedural requirements [21]. Then, IEP team members and students may compile major themes from their answers collaboratively into a format such as a poster that is easy to read. Such an easily accessible format helps diminish communication constraints that occurred in reading and preparing heavy paperwork in traditional IEPs for legal documentation. Reconstructing the planning processes with ease of comprehension and teamwork has been shown to enhance students’ entitlement to their educational planning efforts and school-parental alliance level [18,21].

Overall, a shift from deficit-focused to strengths-based IEPs may significantly deepen teachers’ perception of neurodiversity and make incentives for IEPs internally driven by students’ unique traits. Transforming teachers’ entrenched preference for problem prevention into student empowerment is also a matter of awareness of their unconscious biases and choices to oversee them, regardless of how power dynamics act upon each individual.

Given the current state of IEP research, it is a sub-area of study in special education that is not fully comprehended. Canada is often acknowledged as one of the world leaders for its strong commitment to multiculturalism, equity, inclusion, and diversity in school services and educational outcomes. However, systemic inequities and discriminatory legacies, including racism, colonialism, and ableism, continue to disproportionately affect provincial educational systems and regional school boards to bridge educational gaps and advance inclusive education models [30]. Its relationship with IEP and special education is yet to be substantially investigated to challenge the existing archaic biomedical model and instead adopt a biosocial, action-oriented, and community-based model that aligns with the increasing mental health awareness campaign and knowledge of psychological well-being.

Future direction

This article provides a grounded basis for future research to oversee how strengths-based IEPs help reconstruct collaborative relationships by creating a critical consciousness of mental health literacy and power relationships. However, further research is needed to evaluate the efficacy of strengths-based approaches in engendering meaningful post-school transitions. Moreover, there requires an extensive analysis of systemic barriers to contextualized IEP implementations, both nationally and transnationally. Increasing attention may be put into inspecting the impact of neoliberal education policies on competition among schools, which potentially breed interest-driven IEPs that concentrate on bolstering school reputations. Other systemic concerns involve racial disparity in plan implementations and differential social perceptions of divergent groups. It is also reported that school boards and mental health service providers across Canada (including Ontario) received budget cutbacks for special needs programs for exceptional students despite their very efforts to overcome waitlists for clinical assessment, backlog in file processing, and insufficient provincial funds via accountability campaign and getting through paperwork [31]. Overall, special education is an urgent field of study that necessitates future research.

Limitation

The limitation of this article is that the scope of this brief narrative review does not allow multiscalar and intersectional approaches of analyses on race, gender, class, and legal constitution. Consequently, this article calls for scholars to outline future research on IEPs using an intersectional lens.

## Conclusions

This review article analyzes power dynamics in IEP processes to deconstruct K-12 educators’ judgemental perceptions of exceptional students embedded in hegemonic discourses, as well as for educators to shift their focus to a strengths-based approach. Giving weight to families’ contributions may leverage students' self-determination and transform the IEP emphasis on behavioral prevention into the self-advocacy of personal assets. When collectively reconstructing the special education goals, using a strengths-based approach may challenge educators’ presumptions about their privileges by taking on perspectives from marginalized groups. Through collaborative relationship constructions and teaching mental health literacy, IEP processes may no longer become a school-dominated practice, and students would eventually be stewards of their betterment with more satisfaction toward their individual needs. As a consequence, IEP implementation may become more coherent with students’ strengths and realistic goals for educational benefits and future transition.
